# Phosphodiesterase 3A rs7134375 single nucleotide polymorphism and serum lipid levels

**DOI:** 10.3892/mmr.2014.2007

**Published:** 2014-03-04

**Authors:** WEI WANG, RUI-XING YIN, DONG-FENG WU, LYNN HTET HTET AUNG, PING HUANG, XIAO-NA ZENG, KE-KE HUANG, QUAN-ZHEN LIN, JIAN WU, TAO GUO

**Affiliations:** Department of Cardiology, Institute of Cardiovascular Diseases, The First Affiliated Hospital, Guangxi Medical University, Nanning, Guangxi 530021, P.R. China

**Keywords:** phosphodiesterase 3A gene, single nucleotide polymorphism, lipids, apolipoproteins

## Abstract

The association between the phosphodiesterase 3A (*PDE3A*) rs7134375 single nucleotide polymorphism (SNP) and serum lipid levels are not well understood in the general population. The present study was performed in order to detect the association between the rs7134375 SNP and serum lipid levels in the Guangxi Mulao and Han populations. The genotypes of the *PDE3A* rs7134375 SNP in 761 subjects of the Mulao population and 774 subjects of the Han Chinese population were determined by polymerase chain reaction and restriction fragment length polymorphism combined with gel electrophoresis, and then confirmed by direct sequencing. It was observed that serum low-density lipoprotein cholesterol and apolipoprotein B levels were higher in the Mulao population than in the Han population (P*<*0.05 for each). The frequencies of the C and A alleles were 72.14 and 27.86% in the Mulao population, and 78.55 and 21.45% in the Han population (P<0.01), respectively. The frequencies of the CC, CA and AA genotypes were 52.04, 40.21 and 7.75% in the Mulao population, and 61.50, 34.11 and 4.39% in the Han population (P<0.01), respectively. The frequencies of the C and A alleles were 74.89 and 25.11% in Mulao females, and 68.08 and 31.92% in Mulao males (P<0.01), respectively. The serum triglyceride (TG) levels were different among the genotypes in the Mulao population; however, not in the Han population (P<0.01), and the A allele carriers exhibited lower TG levels than the A allele noncarriers. The serum lipid parameters were also correlated with several environmental factors in the two ethnic groups (P<0.05-0.001). It was concluded that the genotypic and allelic frequencies of the rs7134375 SNP are different between the Mulao and Han populations. In addition, the *PDE3A* rs7134375 SNP is associated with serum TG levels in the Mulao population, however, not in the Han population.

## Introduction

Coronary heart disease (CHD) is the leading cause of mortality and disability worldwide (1). Consistent and compelling evidence has demonstrated the association between dyslipidemia and CHD (2–5). Dyslipidemia, particularly increased serum low-density lipoprotein cholesterol (LDL-C) and triglyceride (TG) levels, is a well-described risk factor for atherosclerosis and CHD (6). It is well established that dyslipidemia is a complex trait caused by multiple environmental and genetic factors and their interactions (7–10). Family studies suggest that in numerous populations, approximately half of the variation in serum lipid profiles is genetically determined (11). Previous genome-wide association studies (GWAS) in different populations have identified >95 loci associated with serum lipid levels (12–22). Common variants at these loci together explain <10% of variation in each lipid trait. Rare variants with large individual effects may also contribute to the heritability of lipid traits (23). Phosphodiesterase 3A (*PDE3A*), a gene located on chromosome 12p12, is a member of the PDE family, and is located in an intron of the gene. PDEs selectively catalyze the hydrolysis of 3′ cyclic phosphate bonds in adenosine and/or guanine 3′, 5′ cyclic monophosphate (cAMP and/or cGMP). They regulate the cellular levels, localization and duration of action of these second messengers by controlling the rate of their degradation. PDE3A has a high affinity for cAMP and cGMP, and demonstrates competitive inhibition of the cAMP hydrolytic activity of cGMP. It is important in regulating intracellular levels of cAMP and cGMP (24–28). The rs7134375 single nucleotide polymorphism (SNP) with an A/C variation in *PDE3A* has been associated with high-density lipoprotein cholesterol (HDL-C) (12,29); however, it remains to be elucidated whether other serum lipid parameters have a connection with it.

China is a multi-ethnic country with 56 ethnic groups. The Han nationality is the largest ethnic group and the Mulao nationality is one of the 55 ethnic minorities with a population of 207,352 according to the fifth national census statistics of China in 2000 (30). The majority of this population have been living in the Luocheng Mulao Autonomous County (Guangxi Zhuang Autonomous Region, China). The history of this minority can be traced back to the Jin Dynasty (AD 265–420). A previous study demonstrated that the genetic association between the Mulao nationality and other minorities in Guangxi was significantly closer than that between the Mulao and Han or Uighur nationality (31). To the best of our knowledge, however, the association between the rs7134375 SNP in *PDE3A* and serum lipid levels has not been previously reported in this population. Therefore, the present study was performed to evaluate the association between the rs7134375 SNP and several environmental factors with serum lipid levels in the Guangxi Mulao and Han populations.

## Materials and methods

### Study population

A total of 761 Mulao subjects who reside in the Luocheng Mulao Autonomous County and 774 Han subjects who reside in the same villages were included in the present study. The subjects of the Mulao population consisted of 307 (40.34%) males and 454 (59.66%) females, ranging between 16 and 93 years old, with a mean age of 51.86±14.82 years. The subjects of the Han population consisted of 286 (36.95%) males and 488 (63.05%) females, aged 16–92 years, with a mean age of 52.05±14.95 years. All the subjects were randomly selected from our previous stratified randomized samples (32,33). All the study subjects were healthy rural agricultural workers and had no evidence of diseases associated with atherosclerosis, CHD or diabetes. The participants were not taking medications known to affect serum lipid levels (for example lipid-lowering drugs, including statins or fibrates, beta-blockers, diuretics, contraceptives or hormones). The present study was approved by the Ethics Committee of the First Affiliated Hospital, Guangxi Medical University (Nanning, China). Informed consent was obtained from all the subjects following receiving a full explanation of the present study.

### Epidemiological survey

The survey was performed using internationally standardized methods (34). Information concerning demographics, socioeconomic status and lifestyle factors was collected with standardized questionnaires. The information collected regarding alcohol intake, included the number of Liangs (~50 g) of rice wine, corn wine, rum, beer or liquor consumed during the preceding 12 months. Alcohol consumption was categorized into two groups depending on the grams of alcohol intake per day: ≤25 and >25. Smoking status was also categorized into groups depending on the number of cigarettes per day: ≤20 and >20. Height, weight and waist circumference were manually measured under the supervision of two individuals. Sitting blood pressure was measured using a mercury sphygmomanometer (Jiangsu Yuwell Medical Equipment and Supply Co., Ltd., Danyang, China). during three separate intervals after a 5 min rest period, and the average of the three measurements was used taken as the blood pressure. Body mass index (BMI) was calculated as the weight in kg divided by the square of height in meters (kg/m^2^).

### Biochemical analysis

Venous blood samples were obtained from all subjects following ≥12 h of fasting. The levels of serum total cholesterol (TC), TG, HDL-C and LDL-C in samples were determined by enzymatic methods with commercially available kits (Tcho-1 and TG-LH; Randox Laboratories Ltd., Crumlin, UK; Cholestest N HDL and Cholestest LDL; Daiichi Pure Chemicals Co., Ltd., Tokyo, Japan, respectively). Serum apolipoprotein (Apo) AI and ApoB levels were detected by the immunoturbidimetric immunoassay using a commercial kit (APO CAL; LP3023; Randox Laboratories Ltd.) (32,33).

### DNA amplification and genotyping

Genomic DNA was extracted from peripheral blood leukocytes using the phenol-chloroform method (32–35). The extracted DNA was stored at 4°C for future analysis. Genotyping of the rs7134375 SNP was performed using a pair of primers: Forward: 5′-TGGGAATCGTTCTTGTTT-3′ and reverse: 5′-GAAAGCCTAAGAGTAATTCATG-3′ (Sangon, Shanghai, China). Each 20 *μ*l PCR reaction mixture consisted of 1 μl genomic DNA, 0.8 μl of each primer (10 pmol/l), 10 μl of 2X *Taq* PCR Mastermix (20 mM Tris-HCl, pH 8.3; 100 mM KCl, 3 mM MgCl_2_, 0.1 units *Taq* Polymerase/μl and 500 μM dNTP each) and 8 μl ddH_2_O (DNase/RNase-free). The reaction mixture was subjected to denaturation at 95°C for 5 min, followed by 33 cycles at 95°C for 45 sec, 51°C for 30 sec, 72°C for 50 sec and a final extension at 72°C for 10 min. Then, the amplification products (5 ml) were digested using 5 units *Msp*I restriction enzyme (Fermentas Co., Burlington, ON, Canada) at 37°C overnight. Following restriction enzyme digestion of the amplified DNA, the genotypes were identified by electrophoresis on 2% agarose gels and visualized with ethidium-bromide staining ultraviolet illumination. The genotypes were scored by an experienced reader blinded to epidemiological data and serum lipid levels. Six samples (CC, CA and AA genotypes in two, respectively) detected by PCR-restriction fragment length polymorphism (RFLP) were also confirmed by direct sequencing. The PCR products were purified by low melting point gel electrophoresis and phenol extraction, and then the DNA sequences were analyzed by Shanghai Sangon Biological Engineering Technology & Services Co., Ltd. (Shanghai, China).

### Diagnostic criteria

The normal values of serum TC, TG, HDL-C, LDL-C, ApoAI and ApoB levels, and the ratio of ApoAI to ApoB in our Clinical Science Experiment Center were 3.10–5.17, 0.56–1.70, 0.91–1.81, 2.70–3.20 mmol/l, 1.00–1.78, 0.63–1.14 g/l and 1.00–2.50, respectively (32,35). Hypertension was diagnosed according to the criteria of the 1999 World Health Organization-International Society of Hypertension Guidelines for the Management of Hypertension (36). Normal weight, overweight and obese were defined as a BMI <24, 24–28 and >28 kg/m^2^, respectively (37).

### Statistical analysis

All statistical analyses were performed using the statistical software package SPSS 13.0 (SPSS, Inc., Chicago, IL, USA). Qualitative variables are expressed as the raw count and percentage. The mean ± standard deviation was used for the presentation of quantitative variables. Genotypic and allelic frequencies were calculated by direct counting and the standard goodness-of-fit test was used to assess the Hardy-Weinberg equilibrium. A χ^2^ test was used to evaluate the difference in genotype distribution and gender ratio between the groups. The difference in general characteristics between the Mulao and Han populations was assessed using Student’s unpaired t-test. The association between the genotypes and serum lipid parameters was examined using analysis of covariance. Gender, age, BMI, blood pressure, alcohol consumption and cigarette smoking were adjusted for the statistical analysis. In order to evaluate the correlation between serum lipid parameters and several environmental factors, multiple linear regression analysis with stepwise modeling was also performed. Two-tailed P*<*0.05 was considered to indicate a statistically significant difference.

## Results

### General characteristics and serum lipid levels

Table I shows the general characteristics and serum lipid parameters of the study populations. As compared with the Han population, the Mulao population had higher serum LDL-C and ApoB levels (P<0.05 for each). No significant differences in the age, height, weight, BMI, waist circumference, blood pressure, serum glucose, TC, TG, HDL-C, ApoAI, ApoB, the ratio of ApoAI to ApoB, the percentages of subjects who smoked cigarettes and the ratio of males to females were identified between the two ethnic groups (P>0.05 for all).

### Results of electrophoresis, genotyping and sequencing

**Figure 1:**
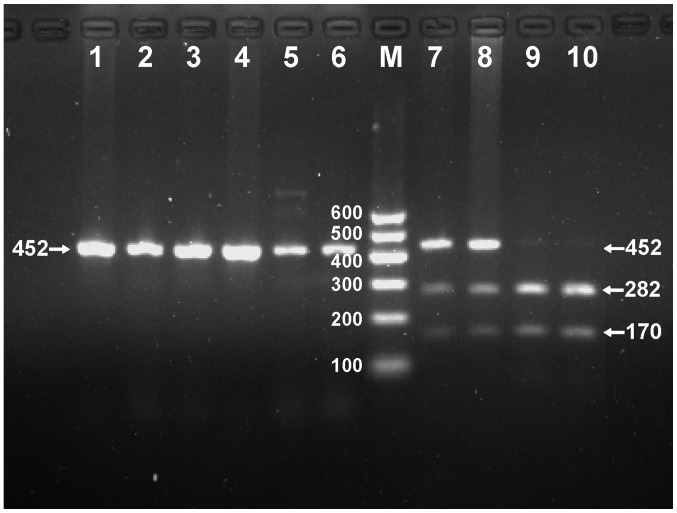
Genotyping of the rs7134375 single nucleotide polymorphism in the phosphodiesterase 3A gene. Lane M, 100 bp marker ladder; lanes 1–4, PCR products (452 bp); lanes 5 and 6, AA genotype (452 bp); lanes 7 and 8, AC genotype (452, 282 and 170 bp), and lanes 9 and 10, CC genotype (282 and 170 bp).

**Figure 2:**
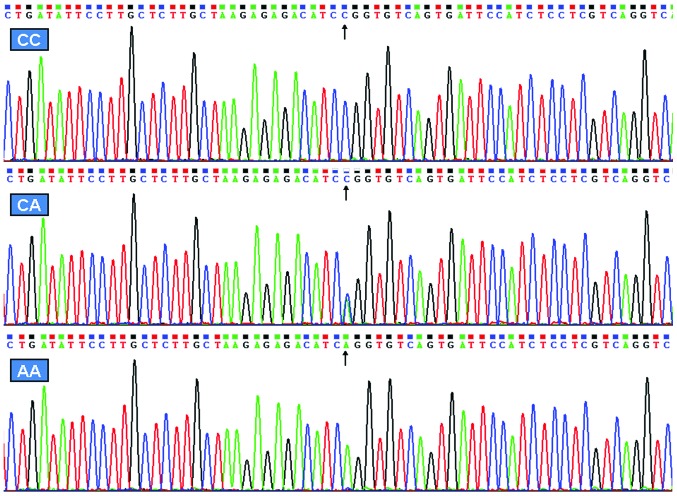
A section of the nucleotide sequence of the rs7134375 single nucleotide polymorphism in the phosphodiesterase 3A gene. The CC, CA and AA genotypes are shown, respectively.

Following amplification of the genomic DNA of the samples by PCR, the PCR products of 452 bp nucleotide sequences were observed in the samples (lanes 1–4; Fig. 1). The genotypes identified were named according to the presence (C allele) or absence (A allele) of the enzyme restriction sites. Thus, the AA genotype is heterozygote for the absence of the site (bands at 452 bp; lanes 5 and 6; Fig. 1), the CA genotype is heterozygote for the absence and presence of the site (bands at 452, 282 and 170 bp; lanes 7 and 8; Fig. 1) and the CC genotype is homozygote for the presence of the site (bands at 282 and 170 bp; lanes 9 and 10; Fig. 1). The AA, CA and CC genotypes detected by the PCR-RFLP were also confirmed by sequencing (Fig. 2), respectively.

### Genotypic and allelic frequencies

The genotypic and allelic frequencies of the *PDE3A* rs7134375 SNP are shown in Table II. The frequency of C and A alleles was 78.55 and 21.45% in the Han population, and 72.14 and 27.86% in the Mulao population, respectively. The frequency of CC, CA and AA genotypes was 61.50, 34.11 and 4.39% in the Han population, and 52.04, 40.21 and 7.75% in the Mulao population, respectively. The genotypic and allelic frequencies were different between the Mulao and Han populations (P<0.01 for each). Significant differences were identified in the genotypic and allelic frequencies between males and females in the Mulao population (P<0.01), however, not in the Han population.

### Genotypes and serum lipid levels

As shown in Table III, the levels of TG in the Mulao population, but not in the Han population, were different among the three genotypes (P<0.05), the A allele carriers exhibited lower TG levels than the A allele noncarriers.

### Risk factors for serum lipid parameters

As shown in Tables IV and V, serum lipid parameters were also correlated with several environmental factors, including age, gender, alcohol consumption, cigarette smoking, blood pressure, blood glucose levels and BMI in the two ethnic groups (P<0.05-0.001).

## Discussion

The present study demonstrated that serum LDL-C and ApoB levels were higher in the Mulao than in the Han population. No significant differences in the levels of TC, TG, HDL-C, ApoAI and the ratio of ApoAI to ApoB between the two ethnic groups were identified. It is well known that dyslipidemia is a complex trait caused by environmental and genetic factors. Family and twin studies suggest that in numerous populations, ~40–60% of the variation in serum lipid profiles is genetically determined (7–11).

Mulao is a relatively conservative minority. Lack of communication with the other ethnic groups, their original life habits and culture are still completely conserved to the present day. Glutinous rice and salted foods are their staple diet. Marriages arranged by parents were common. Prenatal betrothal and cousin marriages were also popular in this minority. Brides did not live with their husbands until the first child was born (38). Therefore, it is considered that the hereditary characteristics and genotypes of certain lipid metabolism-related genes in this population may be different from those in the Han Chinese.

The genotypic and allelic frequencies of the rs7134375 SNP in diverse racial/ethnic groups are different, which can be found on the International HapMap project website (http://hapmap.ncbi.nlm.nih.gov/cgi-perl/gbrowse/hapmap24_B36/#search). The frequency of CC, CA and AA genotypes was 36.7, 43.3 and 20.0% in Utah residents with ancestry from Northern and Western Europe (CEU); 47.5, 42.4 and 10.2% in Yoruba in Ibadan, Nigeria (YRI); 54.5, 45.5 and 0% in Japanese in Tokyo, Japan (JPT) and 53.3, 42.2 and 4.4% in Han Chinese in Beijing, China (CHB). The frequency of C and A alleles was 58.3 and 41.7% in CEU; 68.6 and 31.4% in YRI; 77.3 and 22.7% in JPT and 74.4 and 25.6% in CHB. The present study identified significant differences in the genotypic frequency of the *PDE3A* rs7134375 SNP between the two ethnic groups. The frequency of the CC genotype was lower in the Mulao than in the Han population and the frequency of the C allele was lower in the Mulao than in the Han population (72.14 vs. 78.55%; P*<*0.001). These results suggest that the prevalence of the rs7134375 SNP in *PDE3A* may exhibit a racial/ethnic difference.

The potential association between the rs7134375 SNP and plasma or serum lipid levels in humans has been evaluated in several previous GWAS. The rs7134375 SNP has been associated with serum HDL-C levels (12). At present, no studies have verified that it is associated with other lipid parameters. The current study demonstrated that the A allele carriers in the Mulao population; however, not in the Han population, were associated with lower serum TG levels than the A allele noncarriers. These results suggest that the associations of the rs7134375 SNP in *PDE3A* and serum lipid phenotypes may have a racial/ethnic specificity. cAMP and cGMP are important second messengers involved in intracellular signal transduction in numerous cell types, including vascular smooth muscle cells (VSMCs) (39). The biological processes regulated by cAMP and cGMP in VSMCs include cell contractility, proliferation, migration, apoptosis and inflammatory responses, all of which have been implicated in the regulation of atherogenesis and post angioplasty restenosis (40). Cyclic nucleotide PDEs are important in regulating intracellular cyclic nucleotide (cAMP and cGMP) levels and compartmentalization via degradation of cyclic nucleotides. PDE3 is the major cAMP-hydrolyzing PDE present in VSMCs and oocytes, and its inhibition by nitric oxide-induced accumulation of cGMP results in increased cAMP and protein kinase A activity (41–43). Begum *et al* (44) revealed that therapies specifically aimed at inhibiting the PDE3A isoform may decrease atherosclerosis, and improve metabolic syndrome and post angioplasty restenosis. No clear mechanism on how PDE3 affects the levels of blood lipids was identified; however, it is hypothesized that PDE3 affects blood lipid levels by interfering with the expression of cAMP and cGMP.

Furthermore, exposure to different lifestyles and environments in the populations resident in Guangxi may further modify the effects of genetic variation on blood lipids. The present study demonstrated that serum lipid parameters were correlated with age, gender, alcohol consumption, cigarette smoking, BMI and blood pressure in the two ethnic groups. These data suggest that environmental factors are also important in determining serum lipid levels in populations. Although Mulao and Han nationalities reside in the same region, the diet and lifestyles are different between the two ethnic groups. The individuals of the Mulao nationality are more inclined to eat cold foods along with acidic and spicy dishes. They also enjoy eating animal offal, which contains an abundance of saturated fatty acids. On the contrary, the Han people prefer to eat relatively healthy diets, which include high-quality protein, low-saturated fatty acids, and vitamin-rich vegetables and fruits. The effects of dietary macronutrients on serum lipid levels and their effects on CHD have been extensively studied (45,46). Serum cholesterol concentrations in humans respond more to the major dietary nutrients than to dietary cholesterol itself. Higher serum TC and TG levels have been found in the populations consuming high-saturated fatty acid diets, whereas lower levels of them have been noted in the populations with high carbohydrate or monounsaturated fatty acid diets. Thus, the findings of the present study may be helpful in identifying susceptibility genes, changing unhealthy lifestyles, and preventing environmental risk factors for lipid-related diseases.

In conclusion, the genotypic and allelic frequencies of the rs7134375 SNP were different between the Mulao and Han populations. The associations of the SNP and serum lipid levels were also different between the two ethnic groups. The *PDE3A* rs7134375 SNP was shown to be associated with serum TG levels in the Mulao, however, not in the Han populations. In addition, the A allele carriers were shown to exhibit lower TG levels than the A allele noncarriers.

## Figures and Tables

**Table I:** Comparison of general characteristics and serum lipid levels between Mulao and Han populations.

Parameter	Han	Mulao	t (χ^2^)	P-value
Number	774	761		
Male/female	286/488	307/454	1.861	0.173
Age (years)	52.05±14.95	51.86±14.82	0.253	0.803
Height (cm)	154.24±7.83	155.84±7.89	1.489	0.137
Weight (kg)	53.16±8.73	52.49±9.18	1.466	0.143
Body mass index (kg/m^2^)	22.10±3.35	21.83±3.06	1.645	0.100
Waist circumference (cm)	75.20±7.84	74.86±7.47	0.403	0.416
Cigarette smoking (n %)				
Nonsmoker	584 (75.45)	594 (78.06)		
≤20 cigarettes/day	168 (21.71)	142 (18.66)	2.347	0.309
>20 cigarettes/day	22 (2.84)	25 (3.28)		
Alcohol consumption [n (%)]				
Nondrinker	624 (80.62)	602 (79.11)		
≤25 g/day	72 (9.30)	60 (7.88)	3.867	0.145
>25 g/day	78 (10.08)	99 (13.01)		
Systolic blood pressure (mmHg)	129.11±19.26	128.86±21.40	0.246	0.806
Diastolic blood pressure (mmHg)	81.98±11.19	81.040±11.25	1.637	0.102
Pulse pressure (mmHg)	47.13±14.42	47.82±16.17	0.872	0.383
Glucose (mmol/l)	6.28±1.79	6.19±1.67	0.457	0.511
Total cholesterol (mmol/l)	4.96±1.05	5.08±1.24	1.883	0.060
Triglycerides (mmol/l)	1.39±1.35	1.36±0.94	0.499	0.618
HDL-C (mmol/l)	1.73±0.53	1.77±0.45	1.583	0.114
LDL-C (mmol/l)	2.87±0.87	2.98±0.91	−2.565	0.010
ApoAI (g/l)	1.33±0.26	1.33±0.42	0.183	0.855
ApoB (g/l)	0.85±0.20	0.98±0.54	−6.394	0.000
ApoAI/ApoB	1.65±0.50	1.59±0.94	1.508	0.130

HDL-C, high-density lipoprotein cholesterol; LDL-C, low-density lipoprotein cholesterol; ApoAI, apolipoprotein AI; ApoB, apolipoprotein B.

**Table II:** Comparison of the genotypic and allelic frequencies of the rs7134375 single nucleotide polymorphism between the Mulao and Han populations [n (%)].

		Genotype			Allele	
			
Group	n	CC	CA	AA	C	A
Mulao	761	396 (52.04)	306 (40.21)	59 (7.75)	1098 (72.14)	424 (27.86)
Han	774	476 (61.50)	264 (34.11)	34 (4.39)	1216 (78.55)	332 (21.45)
χ^2^	-	17.046			16.994	
P-value	-	0.000			0.000	
Mulao						
Male	307	148 (48.21)	122 (39.74)	37 (12.05)	418 (68.08)	196 (31.92)
Female	454	248 (54.63)	184 (40.53)	22 (4.84)	680 (74.89)	228 (25.11)
χ^2^	-	13.746			8.457	
P-value	-	0.001			0.004	
Han						
Male	355	193 (54.4)	140 (39.4)	22 (6.20)	526 (74.1)	184 (25.9)
Female	452	252 (55.8)	162 (35.8)	38 (8.4)	666 (73.7)	238 (26.3)
χ^2^	-	2.062			0.035	
P-value	-	0.357			0.852	

**Table III:** Genotypes of the rs7134375 single nucleotide polymorphism and serum lipid levels in the Mulao and Han populations.

Genotype	n	TC (mmol/l)	TG (mmol/l)	HDL-C (mmol/l)	LDL-C (mmol/l)	ApoAI (g/l)	ApoB (g/l)	ApoAI/ApoB
Mulao								
CC	396	5.10±1.34	1.14 (0.87)	1.78±0.48	3.03±0.91	1.35±0.42	0.97±0.51	1.58±0.68
CA	306	5.08±1.15	1.03 (0.66)	1.76±0.42	2.95±0.94	1.31±0.38	0.99±0.58	1.63±1.24
AA	59	4.88±0.86	1.01 (0.97)	1.71±0.45	2.89±0.78	1.27±0.41	0.99±0.54	1.48±0.64
F-value	-	0.870	7.010	0.809	1.911	1.757	0.054	0.635
P-value	-	0.420	0.030	0.445	0.149	0.173	0.947	0.530
CC	396	5.10±1.34	1.14 (0.87)	1.78±0.48	3.03±0.91	1.35±0.42	0.97±0.51	1.58±0.68
CA/ AA	365	5.05±1.11	1.02 (0.66)	1.75±0.42	2.94±0.94	1.30±0.39	0.99±0.56	1.61±1.17
F-value	-	0.638	2.605	0.980	1.303	1.783	−0.313	−0.331
P-value	-	0.524	0.009	0.327	0.193	0.075	0.755	0.741
Han								
CC	476	4.97±1.02	1.03 (0.90)	1.71±0.42	2.84±0.84	1.34±0.26	0.85±0.21	1.65±0.47
CA	264	4.93±1.09	1.06 (0.71)	1.76±0.69	2.82±0.92	1.32±0.27	0.83±0.21	1.67±0.55
AA	34	5.17±1.10	1.22 (0.97)	1.71±0.46	3.12±0.80	1.35±0.23	0.91±0.13	1.50±0.30
F-value	-	0.797	1.372	0.613	1.686	0.676	2.352	1.751
P-value	-	0.451	0.503	0.542	0.186	0.509	0.096	0.174
CC	476	4.97±1.02	1.03 (0.90)	1.71±0.42	2.84±0.84	1.34±0.26	0.85±0.21	1.65±0.47
CA/ AA	298	4.96±1.09	1.08 (0.73)	1.75±0.67	2.91±0.91	1.33±0.27	0.84±0.20	1.65±0.53
F-value	-	0.011	−0.231	−0.998	−0.967	0.973	0.604	−0.001
P-value	-	0.915	0.817	0.318	0.334	0.331	0.546	0.999
Mulao/male								
CC	148	4.98±1.64	1.36±0.97	1.78±0.53	2.82±0.89	1.34±0.43	0.99±0.67	1.65±0.78
CA/ AA	159	4.72±1.07	1.17±0.83	1.70±0.44	2.73±0.88	1.31±0.40	0.91±0.53	1.82±1.60
F-value	-	1.684	1.821	1.356	0.862	0.629	1.138	−1.186
P-value	-	0.093	0.070	0.176	0.389	0.530	0.256	0.237
Mulao/female								
CC	248	5.17±1.12	1.47±0.99	1.78±0.45	3.15±0.90	1.36±0.41	0.96±0.39	1.54±0.60
CA/ AA	206	5.30±1.08	1.36±0.93	1.78±0.40	3.10±0.91	1.30±0.38	1.05±0.58	1.44±0.60
F-value	-	−1.194	1.243	−0.027	0.582	1.789	−1.723	1.837
P-value	-	0.233	0.214	0.978	0.561	0.074	0.085	0.067
Han/male								
CC	172	4.94±1.00	1.47±1.10	1.68±0.41	2.85±0.85	1.30±0.25	0.85±0.20	1.62±0.47
CA/ AA	114	4.99±1.06	1.37±1.00	1.70±0.41	2.93±0.91	1.29±0.25	0.85±0.21	1.62±0.58
F-value	-	−0.376	0.808	−0.381	−0.758	0.340	−0.060	−0.034
P-value	-	0.707	0.420	0.704	0.449	0.734	0.952	0.973
Han/female								
CC	304	4.98±1.03	1.025 (0.760)	1.73±0.42	2.84±0.84	1.36±0.25	0.85±0.21	1.67±0.47
CA/ AA	184	4.94±1.12	1.025 (0.760)	1.78±0.79	2.89±0.90	1.33±0.28	0.84±0.20	1.67±0.51
F-value	-	0.413	−0.371	−0.972	−0.626	0.896	0.803	−0.014
P-value	-	0.680	0.710	0.332	0.532	0.371	0.422	0.989

TC, total cholesterol; TG, triglyceride; HDL-C, high-density lipoprotein cholesterol; LDL-C, low-density lipoprotein cholesterol; ApoAI, apolipoprotein AI; ApoB, apolipoprotein B. The value for TG is presented as the median (interquartile range). The difference between the two ethnic groups was determined by the Wilcoxon-Mann-Whitney test.

**Table IV:** Association between serum lipid parameters and relative factors in the Mulao and Han populations.

A, Han and Mulao						

Lipid parameter	Risk factor	Unstandardized Coefficient.	Std error	Standardized coefficient	t	P-value
TC	Age	0.013	0.002	0.163	6.359	0.000
	Waist circumference	0.016	0.004	0.113	4.379	0.000
	Alcohol consumption	0.003	0.001	0.100	3.985	0.000
	Diastolic blood pressure	0.006	0.003	0.055	2.053	0.040
	Ethnic group	−0.112	0.057	−0.049	−1.977	0.048
TG	Waist circumference	0.041	0.004	0.287	11.556	0.000
	Systolic blood pressure	0.004	0.001	0.068	2.749	0.006
	Alcohol consumption	0.002	0.001	0.065	2.677	0.008
HDL-C	Waist circumference	−0.014	0.002	−0.234	−9.375	0.000
	Alcohol consumption	0.002	0.000	0.098	3.949	0.000
	Age	0.001	0.001	0.065	2.628	0.009
LDL-C	Age	0.011	0.001	0.183	7.426	0.000
	Body mass index	0.032	0.009	0.117	3.433	0.001
	Ethnic group	−0.131	0.044	−0.073	−2.979	0.003
	Waist circumference	0.009	0.004	0.085	2.500	0.013
ApoAI	Alcohol consumption	0.002	0.000	0.201	8.014	0.000
	Waist circumference	−0.004	0.001	−0.100	−3.976	0.000
ApoB	Waist circumference	0.010	0.001	0.207	8.150	0.000
	Ethnic group	−1.134	0.020	−0.163	−6.655	0.000
	Systolic blood pressure	0.003	0.001	0.160	4.874	0.000
	Diastolic blood pressure	−0.003	0.001	−0.092	−2.763	0.006
ApoAI/ApoB	Waist circumference	−0.020	0.002	−0.212	−8.461	0.000
	Age	−0.005	0.001	−0.102	−4.101	0.000
	Alcohol consumption	0.001	0.001	0.049	1.975	0.048

B, Han						

Lipid parameter	Risk factor	Unstandardized Coefficient	Std. error	Standardized coefficient	t	P-value

TC	Diastolic blood pressure	0.014	0.003	0.151	4.154	0.000
	Waist circumference	0.022	0.005	0.164	4.702	0.000
	Age	0.010	0.002	0.145	3.960	0.000
	Alcohol consumption	0.004	0.001	0.140	4.113	0.000
	Glucose	0.052	0.021	0.085	2.438	0.015
TG	Waist circumference	0.055	0.006	0.318	9.389	0.000
	Glucose	0.083	0.026	0.106	3.162	0.002
	Alcohol consumption	0.004	0.001	0.100	2.941	0.003
HDL-C	Waist circumference	−0.013	0.002	−0.185	−5.192	0.000
	Alcohol consumption	0.001	0.001	0.073	2.042	0.041
LDL-C	Age	0.011	0.002	0.201	5.634	0.000
	Waist circumference	0.012	0.005	0.111	2.411	0.016
	Body mass index	0.028	0.012	0.109	2.371	0.018
	Glucose	0.041	0.018	0.082	2.308	0.021
ApoAI	Alcohol consumption	0.002	0.000	0.288	8.388	0.000
	Body mass index	−0.010	0.003	−0.124	−3.594	0.002
	Cigarette smoking	0.038	0.013	0.070	2.035	0.042
	Gender	0.094	0.024	0.080	3.549	0.000
ApoB	Waist circumference	0.006	0.001	0.222	4.397	0.000
	Glucose	0.023	0.004	0.192	5.586	0.000
	Alcohol consumption	0.048	0.011	0.152	4.430	0.000
	Systolic blood pressure	0.001	0.000	0.133	3.823	0.000
	Body mass index	0.010	0.003	0.143	2.837	0.005
ApoAI/ApoB	Waist circumference	−0.013	0.003	−0.198	−4.352	0.000
	Gender	−0.006	0.001	−0.099	−2.824	0.005
	Body mass index	−0.015	0.007	−0.171	−3.780	0.000
	Glucose	−0.026	0.010	−0.090	−2.588	0.010
	Alcohol consumption	0.001	0.001	0.068	2.011	0.045

C, Mulao						

Lipid parameter	Risk factor	Unstandardized Coefficient	Std. error	Standardized coefficient	t	P-value

TC	Age	0.015	0.003	0.181	5.083	0.000
	Alcohol consumption	0.002	0.001	0.074	2.087	0.037
TG	Waist circumference	0.030	0.004	0.268	7.514	0.000
	Systolic blood pressure	0.004	0.002	0.090	2.521	0.012
HDL-C	Body mass index	−0.023	0.008	−0.156	−3.018	0.003
	Alcohol consumption	0.001	0.000	0.114	3.277	0.001
	Waist circumference	−0.009	0.003	−0.171	−3.276	0.001
	Age	0.002	0.001	0.076	2.194	0.029
LDL-C	Gender	0.341	0.065	0.183	5.219	0.000
	Body mass index	0.055	0.010	0.182	5.203	0.000
ApoAI	Alcohol consumption	0.002	0.000	0.157	4.388	0.000
	Waist circumference	−0.009	0.003	−0.190	−3.535	0.000
	Body mass index	0.015	0.007	0.113	2.188	0.034
ApoB	Waist circumference	0.012	0.001	0.188	5.121	0.000
	Systolic blood pressure	0.005	0.002	0.184	3.851	0.000
	Diastolic blood pressure	−0.018	0.001	−0.162	−3.367	0.001
ApoAI/ApoB	Waist circumference	−0.020	0.004	−0.183	−4.955	0.000
	Age	−0.008	0.002	−0.131	−3.572	0.000
	Diastolic blood pressure	0.006	0.003	0.077	2.038	0.042

TC, total cholesterol; TG, triglyceride; HDL-C, high-density lipoprotein cholesterol; LDL-C, low-density lipoprotein cholesterol; ApoAI, apolipoprotein AI; ApoB, apolipoprotein B.

**Table V:** Association between serum lipid parameters and relative factors in males and females of the Mulao and Han populations.

A, Han/male						

Lipid parameter	Risk factor	Unstandardized Coefficient	Std. error	Standardized coefficient	t	P-value
TC	Age	0.014	0.004	0.213	3.677	0.000
	Waist circumference	0.021	0.008	0.164	2.824	0.005
	Diastolic blood pressure	0.013	0.005	0.143	2.385	0.018
TG	Waist circumference	0.049	0.007	0.361	6.557	0.000
	Alcohol consumption	0.004	0.002	0.114	2.073	0.039
HDL-C	Waist circumference	−0.012	0.003	−0.222	−3.838	0.000
LDL-C	Age	0.013	0.003	0.239	4.064	0.000
	Body mass index	0.044	0.016	0.158	2.783	0.006
	Glucose	0.058	0.028	0.121	2.048	0.041
ApoAI	Alcohol consumption	0.002	0.000	0.241	4.158	0.000
ApoB	Waist circumference	0.008	0.001	0.294	5.283	0.000
	Diastolic blood pressure	0.001	0.001	0.133	2.161	0.032
	Glucose	0.013	0.006	0.116	2.040	0.042
ApoAI/ApoB	Waist circumference	−0.016	0.004	−0.249	−4.356	0.000
	Age	−0.004	0.002	−0.124	−2.165	0.031

B, Han/female						

Lipid parameter	Risk factor	Unstandardized Coefficient	Std. error	Standardized coefficient	t	P-value

TC	Diastolic blood pressure	0.014	0.004	0.146	3.180	0.002
	Waist circumference	0.024	0.006	0.177	4.052	0.000
	Alcohol consumption	0.006	0.001	0.187	4.350	0.000
	Age	0.010	0.003	0.145	3.265	0.001
TG	Waist circumference	0.058	0.008	0.302	7.016	0.000
	Glucose	0.115	0.032	0.128	3.016	0.003
	Alcohol consumption	0.004	0.002	0.092	2.157	0.032
HDL-C	Waist circumference	−0.012	0.003	−0.158	−3.532	0.000
LDL-C	Waist circumference	0.024	0.005	0.217	4.977	0.000
	Age	0.010	0.002	0.185	4.248	0.000
ApoAI	Alcohol consumption	0.002	0.062	0.307	7.120	0.000
	Body mass index	−0.010	0.000	−0.130	−3.017	0.003
ApoB	Waist circumference	0.020	0.003	0.312	7.302	0.000
	Systolic blood pressure	0.021	0.005	0.178	4.011	0.000
	Alcohol consumption	0.003	0.001	0.258	5.633	0.000
	Glucose	−0.150	0.047	−0.142	−3.208	0.000
ApoAI/ApoB	Waist circumference	−0.014	0.003	−0.228	−4.168	0.000
	Body mass index	−0.024	0.008	−0.168	−3.099	0.002
	Glucose	−0.028	0.013	−0.095	−2.176	0.030
	Age	−0.003	0.001	−0.093	−2.137	0.033

C, Mulao/male						

Lipid parameter	Risk factor	Unstandardized Coefficient	Std. error	Standardized coefficient	t	P-value

TC	Age	0.036	0.010	0.195	3.422	0.001
	Waist circumference	0.020	0.010	0.115	2.019	0.044
TG	Waist circumference	0.036	0.006	0.316	5.842	0.000
	Alcohol consumption	0.010	0.001	0.123	2.267	0.024
HDL-C	Waist circumference	−0.018	0.003	−0.299	−5.296	0.000
	Age	0.007	0.004	0.114	2.030	0.043
LDL-C	Body mass index	0.051	0.016	0.181	3.149	0.002
	Age	0.016	0.007	0.135	2.351	0.019
ApoAI	Age	0.010	0.003	0.185	3.310	0.001
	Alcohol consumption	0.002	0.001	0.146	2.606	0.030
ApoB	Waist circumference	0.019	0.004	0.246	4.435	0.000
ApoAI/ApoB	Waist circumference	−0.020	0.009	−0.123	−2.167	0.031

D, Mulao/female						

Lipid parameter	Risk factor	Unstandardized Coefficient	Std. error	Standardized coefficient	t	P-value

TC	Age	0.019	0.005	0.192	3.849	0.000
TG	Waist circumference	0.025	0.014	0.231	4.975	0.000
	Systolic blood pressure	0.005	0.005	0.114	2.451	0.015
HDL-C	Body mass index	−0.043	0.006	−0.302	−6.758	0.000
	Alcohol consumption	0.001	0.000	0.12	2.796	0.005
LDL-C	Body mass index	0.050	0.014	0.164	3.530	0.000
ApoAI	Waist circumference	−0.007	0.002	−0.160	−3.459	0.001
	Alcohol consumption	0.001	0.001	0.141	3.065	0.002
ApoB	Waist circumference	0.008	0.003	0.143	3.075	0.002
	Cigarette smoking	1.197	0.340	0.172	3.526	0.000
ApoAI/ApoB	Waist circumference	−0.020	0.003	−0.286	−6.159	0.000
	Age	−0.009	0.003	−0.133	−2.935	0.004
	Diastolic blood pressure	0.006	0.002	0.109	2.316	0.021

TC, total cholesterol; TG, triglyceride; HDL-C, high-density lipoprotein cholesterol; LDL-C, low-density lipoprotein cholesterol; ApoAI, apolipoprotein AI; ApoB, apolipoprotein B.
